# Nature-Inspired Redox Shuttle with Regenerable Antioxidant for Efficient All-Perovskite Tandem Solar Cells

**DOI:** 10.1007/s40820-025-02006-6

**Published:** 2026-01-05

**Authors:** Rui Meng, Liming Du, Can Li, Zhi Wan, Jishan Shi, Yueying Zhang, Wenfeng Liu, Chongyang Zhi, Chunmei Jia, Lili Tan, Chuanxiao Xiao, Xian-Zong Wang, Lin Song, Xingyu Gao, Zhen Li

**Affiliations:** 1 Shenzhen Research Institute of Northwestern Polytechnical University, Sanhang Science & Technology Building, No.45th, Gaoxin South 9th Road, Nanshan District, Shenzhen City, 518057 People’s Republic of China; 2 State Key Laboratory of Solidification Processing, Center for Nano Energy Materials, School of Materials Science and Engineering, Northwestern Polytechnical University and Shaanxi Joint Laboratory of Graphene (NPU), Xi’an, 710072 People’s Republic of China; 3 Ningbo Institute of Materials Technology and Engineering, Chinese Academy of Sciences, Ningbo, 315201 People’s Republic of China; 4 Ningbo New Materials Testing and Evaluation Center CO., Ltd, Ningbo, 315201 People’s Republic of China; 5 Center of Advanced Lubrication and Seal Materials, Northwestern Polytechnical University, Xi’an, 710072 People’s Republic of China; 6 State Key Laboratory of Flexible Electronics (LOFE) & Institute of Flexible Electronics (IFE), Northwestern Polytechnical University, 127 West Youyi Road, Xi’an, 710072 People’s Republic of China; 7 Shanghai Synchrotron Radiation Facility (SSRF), Zhangjiang Laboratory, Shanghai Advanced Research Institute, Chinese Academy of Sciences, 239 Zhangheng Road, Shanghai, 201204 People’s Republic of China

**Keywords:** Pb–Sn perovskite, Redox shuttle, Crystallization regulation, All-perovskite tandem, Stability

## Abstract

Pb–Sn mixed perovskite solar cells (PSCs) are crucial components for realizing efficient all-perovskite tandem devices. However, their efficiency and stability are severely limited by oxidative degradation (Sn^4+^ formation) and metallic defects (Sn^0^/Pb^0^). In addition, the rapid and uncontrolled Sn^2+^ nucleation kinetics result in nonuniform crystallization. Herein, we introduce a natural redox shuttle glutathione (GSH) in Pb–Sn mixed PSCs, achieving regenerable antioxidation and crystallization regulation simultaneously. The reversible redox reactions between GSH and glutathione disulfide (GSSG) enable the self-healing of Sn^4+^ and Sn^0^/Pb^0^ impurities, creating a regenerable antioxidation protective shell at the perovskite interfaces. Meanwhile, the strong coordination between GSH and perovskite regulates the crystallization process, optimizing the nucleation and crystallization kinetics. Furthermore, the GSH incorporation creates a high-quality charge separation junction at the perovskite/hole transport layer, facilitating carrier separation and extraction. The optimized Pb–Sn PSCs exhibit impressive power conversion efficiencies (PCEs) of up to 23.71%. The champion all-perovskite tandem PSCs with GSH achieve a PCE of 28.49% and retain 90% of the initial PCE after 560 h of continuous illumination. This work establishes a new nature-inspired redox shuttling strategy and elucidates its working mechanism, advancing the development of efficient and stable all-perovskite tandem solar cells.

**Supplementary Information:**

The online version contains supplementary material available at 10.1007/s40820-025-02006-6.

## Highlights

- A natural and regenerable redox shuttle is established using glutathione (GSH) to eliminate harmful Sn^4+^ and Sn^0^/Pb^0^ impurities.
- The GSH incorporation regulates the perovskite crystallization process and leads to the formation of a high-quality charge separation junction.
- The GSH-modified Pb-Sn perovskite solar cells achieve a champion power conversion efficiency (PCE) of 23.71%. Furthermore, the resulting all-perovskite tandem solar cells exhibit a PCE of 28.49% and retain 90% of the initial PCE after 560 h of continuous operation.

**Supplementary Information:**

The online version contains supplementary material available at 10.1007/s40820-025-02006-6.

## Graphical abstract

**Figure:**
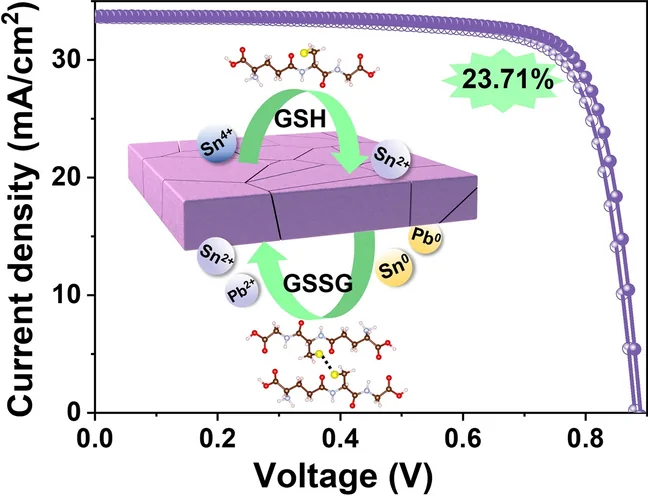

## Introduction

All-perovskite tandem solar cells (TSCs) have emerged as promising candidates to break the Shockley–Queisser (SQ) efficiency limit of single-junction solar cells, while maintaining the intrinsic advantages of perovskite materials, such as low cost, compatibility with flexible substrates and solution processability [1, 2]. Recently, their power conversion efficiencies (PCEs) have reached 30.1% [3], demonstrating substantial commercial potential [4]. However, compared with other TSC technologies, the development of all-perovskite TSCs is still primarily impeded by the performance limitations of Pb–Sn mixed perovskite solar cells (PSCs) [5, 6]. These challenges mainly stem from the easy oxidation of Sn^2+^ to Sn^4+^, resulting in numerous Sn vacancies [7]. Meanwhile, the disproportionation reaction of Sn^2+^ and perovskite degradation introduce unfavorable impurities of Sn^4+^, Sn^0^, and Pb^0^, compromising the optoelectronic properties and device stability [8]. In addition, compared with pure Pb-based perovskite, Sn^2+^ ions exhibit weaker coordination with common solvents such as dimethyl sulfoxide (DMSO) and dimethylformamide (DMF), making them more easily released from the solvent complex, which leads to rapid nucleation and growth of Sn-rich domains. This rapid crystallization process restricts the time available for ion diffusion and lattice reordering, ultimately resulting in the formation of structural defects, such as vacancies, grain boundaries, and pinholes [9]. These defects lead to severe non-radiative recombination, thereby hindering the carrier transport and limiting the photovoltaic performance [10]. Addressing these challenges requires effective strategies to mitigate the redox impurities and modulate the perovskite growth, thereby enhancing the optoelectronic properties and film quality of Pb–Sn perovskite to accelerate the developments of all-perovskite TSCs [11].

Various antioxidant and reducing agents, such as metallic Sn powder [12], ascorbic acid [13], caffeic acid [14], organic hydrazine derivatives [15], and phosphonic acid ligands [16], have been employed to mitigate Sn^2+^ oxidation in Pb–Sn mixed PSCs. These strategies typically function through modulating the redox equilibria and stabilizing metallic intermediate phases, effectively suppressing Sn^2+^ oxidation and alleviating p-type self-doping. However, these approaches normally only address the initial Sn^2+^ oxidation issue, yet unable to eliminate the disproportionation reaction products that formed during device operation. Furthermore, their effectiveness is inherently limited by their consumptive nature in redox reactions, becoming inactive once depleted due to the low concentration in the PSCs. Recently, rosin acid featuring reversible conjugated double bonds was reported to sustainably eliminate degradation products generated during device operation, thereby enabling self-healing [17]. However, there remains a significant need to explore novel regenerable antioxidant materials for eliminating the redox impurities within Pb–Sn PSCs [18].

Beyond the issues of Sn^2+^ oxidation and disproportionation, the perovskite films quality and device performance are significantly compromised by the nonuniform nucleation and crystallization induced by disparate solvent affinity between Sn^2+^ and Pb^2+^ [19]. To address this challenge, molecular engineering strategies have been employed to develop functional additives for regulating perovskite nucleation. For instance, non-covalent binders such as p-phenylenediamine (PPD) achieve synchronized crystallization kinetics through selective chelation of Sn^2+^ [20], while dynamic coordination molecules like N-(carboxyphenyl)guanidine hydrochloride (CPGCl) suppress disordered crystallization by modulating precursor release rates [21]. Recently, amino acid derivatives (such as L-phenylalanine hydrochloride [22] and aspartic acid hydrochloride [23]) have been engineered to establish molecular networks through multidentate coordination, achieving integrated suppression of crystallization kinetics, defect passivation, and structural stabilization. Despite these advances, there remains an urgent need to develop multifunctional additives capable of simultaneously reducing redox impurity and modulating crystallization, thereby advancing both the efficiency and stability of Pb–Sn PSCs and all-perovskite TSCs.

Glutathione (GSH), one of the most abundant biological antioxidants [24], plays a significant role in scavenging free radicals and peroxides within living organisms. Its versatile applications span across diverse fields, including chemical synthesis, clinical medicine [25], food preservation [26], and cosmetic antiaging treatments [27]. In biological systems, GSH functions via the GSH-Glutathione disulfide (GSSG) redox shuttle mechanism, as illustrated in Fig. S1. Under oxidative conditions, the sulfhydryl groups from two GSH molecules form a disulfide bond, producing GSSG [28]. The oxidized form GSSG is subsequently reduced back to GSH by glutathione reductase, utilizing reduced nicotinamide adenine dinucleotide phosphate (NADPH) as an electron donor [29]. Inspired by this biological reversible redox agent, we propose that GSH is expected to function as a regenerable antioxidant for eliminating redox impurities in Pb–Sn perovskite. Additionally, the abundant carbonyl and carboxyl functional groups in GSH can form strong coordination with Sn^2+^, regulating the Pb^2+^/Sn^2+^ crystallization process.

In this work, we demonstrate a biological redox shuttle GSH for Pb–Sn PSC that simultaneously eliminates redox impurities and modulates perovskite crystallization kinetics. The reversible redox reactions between GSH and GSSG in perovskite film enable a regenerable antioxidant mechanism, effectively removing undesirable redox and disproportional decomposition products of Sn^4+^ and Sn^0^/Pb^0^. Furthermore, the strong coordination interactions between GSH and Sn^2+^/Pb^2+^ ions regulate the perovskite crystallization process, optimizing nucleation and growth kinetics. The resultant perovskite film exhibits enhanced crystallinity with preferential orientation along the (100) crystal plane. In addition, GSH at perovskite buried interface and top surface forms high-quality charge separation junction at the buried interface and improves energy level alignment at the perovskite/ETL interface. The enhancements promote efficient carrier separation and extraction at both interfaces. Benefiting from the enhanced optoelectronic properties and superior crystal quality, the Pb–Sn PSCs with GSH redox shuttle achieve an impressive PCE of 23.71% with a high open-circuit voltage of 0.89 V. Further integration into two-terminal (2T) all-perovskite TSC yields a champion PCE of 28.49%. Additionally, the GSH incorporation significantly enhances the device stability due to the recyclable elimination of impurities during the device operation. Specifically, the 2T all-perovskite TSCs retained 90% of the initial PCE after 560 h operation in N_2_ atmosphere, whereas the PCE of PSCs without GSH declined to 48% under identical conditions. Our work introduces an effective strategy of stabilizing Pb–Sn mixed perovskite utilizing biological redox shuttle system and provides insights into the mechanisms in PSCs. The improvements in optoelectronic properties and stability advance the development of Pb–Sn perovskite and all-perovskite tandem cells.

## Experimental Section

### Materials

Tin (II) iodide (SnI_2_, 99.99%), tin (II) fluorine (SnF_2_, 99%), glutathione (GSH), ethylenediamine (EDA, 99%), N,N-dimethylformamide (DMF, 99.9%), dimethyl sulfoxide (DMSO, 99.9%), chlorobenzene (CB, 99.9%) and isopropanol (IPA, 99.5%), and ethyl acetate (EA, 99.8%) were purchased from Sigma-Aldrich. Lead (II) iodide (PbI_2_, 99.99%), lead (II) bromide (PbBr_2_, 99.99%), [4-(3,6-dimethyl-9*H*-carbazol-9-yl)butyl]phosphonic acid (Me-4PACz), and guanidine thiocyanate (GuaSCN, 99%) were purchased from TCI Shanghai Chemical Industry Materials Corp. Methylammonium iodide (MAI, 99.9%) and formamidine iodide (FAI, 99.9%) were purchased from Greatcell Solar Materials Pty Ltd. PEDOT:PSS (CLEVIOS P VP AI 4083) was purchased from Heraeus. NiO_X_ nanoparticle powder (particle size around 10 nm) and Glass/ITO (8 Ω sq^−1^) were purchased from Advanced Election Technology Company in China. Cesium iodide (CsI, 99%), C_60_, Poly[bis(4-phenyl)(2,4,6-trimethylphenyl)amine] (PTAA) and 2-ThEABr were purchased from Xi’an Polymer Light Technology. BCP was purchased from Luminescence Technology Corp. The indium oxide (InO_X_) particles were purchased from Beijing Lichengxin Material Technology Corp. The indium tin oxide (ITO, In_2_O_3_/SnO_2_ 90/10 wt%) target was purchased from Hebei Jiuyue Material Technology Corp. All reagents were used as received without any further purification.

### Device Fabrication

The narrow-bandgap perovskite precursor (FA_0.7_MA_0.3_Sn_0.5_Pb_0.5_I_3_, 2.0 M) was formulated in a mixed solvent of DMF/DMSO (3:1, v/v) with SnF_2_ (0.1 mmol) and GuaSCN (0.04 mmol) as additives. For GSH-incorporated devices, 1 mol% GSH relative to SnI_2_ was introduced. The solution was stirred overnight before film deposition. Pre-patterned ITO substrates were cleaned and plasma-treated, followed by spin-coating of diluted PEDOT:PSS (in IPA, 1:1 v/v) at 4000 rpm for 30 s and annealing at 150 °C for 10 min. The perovskite layer was deposited via a two-step spin-coating process (1000 rpm for 10 s, then 4000 rpm for 40 s), with 200 µL ethyl acetate antisolvent dripping during the second step. The films were annealed sequentially at 60 °C for 2 min and 100 °C for 10 min. Then, 0.1 mM EDA in chlorobenzene was spin-coated at 4000 rpm for 30 s and annealed at 100 °C for 5 min. Finally, C_60_ (40 nm), BCP (7 nm), and Ag (100 nm) were thermally evaporated under high vacuum.

For the wide-bandgap perovskite devices (FA_0.8_Cs_0.2_PbI_1.8_Br_1.2_, 1.2 M), the precursor was dissolved in DMF/DMSO (4:1, v/v) with Pb(SCN)_2_ and PbCl_2_ additives and stirred at 55 °C for 2 h. ITO substrates were cleaned and plasma-treated. A NiO_X_ solution (15 mg mL^−1^ in water) was spin-coated at 4000 rpm for 20 s and annealed at 100 °C for 5 min. Then, Me-4PACz (1 mg mL^−1^ in IPA) was spin-coated at 5000 rpm for 25 s and annealed at 100 °C for 5 min. The perovskite layer was deposited at 5000 rpm for 40 s with ethyl acetate antisolvent dripping at the last 15 s, followed by annealing at 110 °C for 20 min. Subsequently, 2-ThEABr (5 mg mL^−1^ in IPA) was spin-coated at 5000 rpm for 30 s and annealed at 100 °C for 3 min. Finally, C_60_ (30 nm), BCP (5 nm), and Ag (100 nm) were thermally evaporated.

The 2T all-perovskite tandem devices were fabricated by first constructing the wide-bandgap subcell up to the C_60_/BCP layer. Then, a 20 nm InO_X_ interlayer was deposited via electron beam evaporation at 0.05 Å s^−1^, followed by the sputtering of a 100 nm ITO layer (120 W, 0.45 Pa Ar pressure). The narrow-bandgap subcell was subsequently fabricated on top of the ITO recombination layer by repeating the PEDOT:PSS, perovskite, and evaporation steps (C_60_, BCP, Ag) as described for the single-junction narrow-bandgap device.

### Characterization

The perovskite films were characterized using field emission scanning electron microscopy (SEM, ZEISS Sigma 300) at 3 kV. X-ray diffraction (XRD) patterns were collected on a Bruker D8 Discover A25 system with Cu K*α* radiation (λ = 1.5406 Å). X-ray photoelectron spectroscopy (XPS) was performed on a PHI 5000 VersaProbe III instrument, with C 1*s* referenced to 284.6 eV. Fourier transform infrared (FTIR) spectra were acquired using a Shimadzu-IRTracer 100 spectrometer. In situ UV–Vis and steady-state photoluminescence (PL) spectra were recorded using an Ocean Optics NIRQuest 512 spectrometer, with PL excited at 405 nm. Grazing-incidence wide-angle X-ray scattering (GIWAXS) measurements were conducted at the Shanghai Synchrotron Radiation Facility (BL14B1/BL17B1) using a 10 keV X-ray source. Ultraviolet photoelectron spectroscopy (UPS) was carried out on a Kratos Axis Supra system with a He I source (21.22 eV) and a -9.0 V bias. Nuclear magnetic resonance (^1^H NMR) spectra were obtained using a Bruker Advance 400 spectrometer.

The photovoltaic performance of devices was evaluated under AM 1.5 G illumination (100 mW cm^−2^) from a Newport Sol3A solar simulator within a N_2_ glovebox. The light intensity was calibrated using a KG-5 filtered silicon reference cell. Current density–voltage (*J-V*) curves were measured with a Keithley 2400 source meter at a scan rate of 0.1 V s^−1^ from -0.05 to 0.9 V. The device area was defined as 0.032 cm^2^ by a metal aperture mask. External quantum efficiency (EQE) spectra were collected from 300 to 1100 nm using an Enli Tech QE-R 3011 system. For tandem devices, bias light with 900 nm and 550 nm filters was used to measure the subcell responses. Dark *J-V* characteristics were obtained using a Keysight 2400 source meter. Steady-state power output, light-intensity-dependent *V*_*OC*_, and capacitance–voltage (C-V) profiles (100 kHz, 5 mV amplitude) were also measured. Transient photovoltage (TPV) and transient photocurrent (TPC) decays were recorded with a RIGOL DG812 oscillator under 1 sun LED illumination following excitation by a 532 nm pulsed laser. Operational stability tests were performed under continuous 1 sun illumination (100 mW cm^−2^) from a white LED at open-circuit conditions in N_2_ atmosphere at 25 °C using a PURI2400-E8 test system.

## Results and Discussion

### Working Mechanism of the Regenerable GSH-GSSG Redox Shuttle

**Fig. 1:**
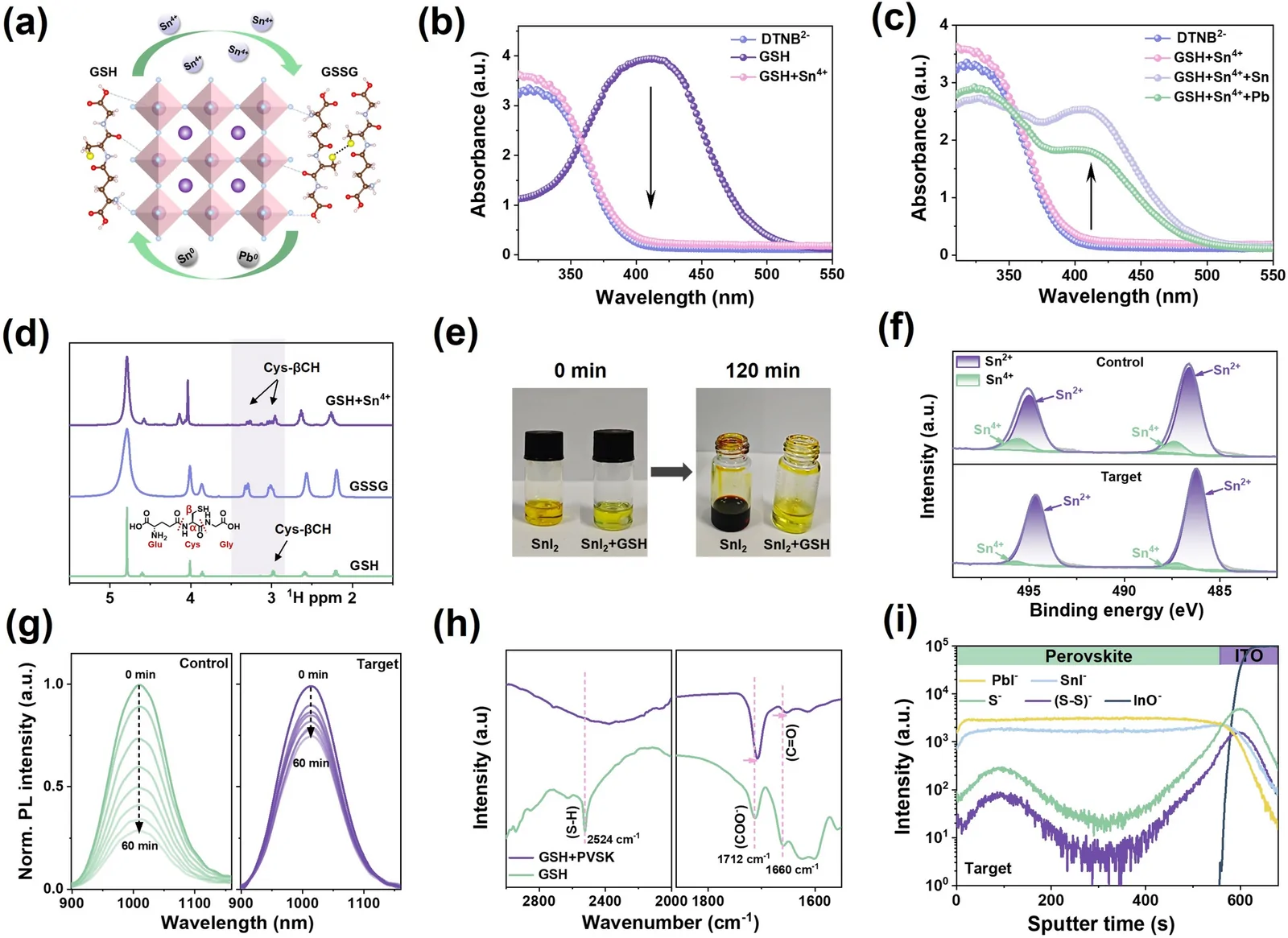
Redox reactions of Sn^2+^ and Sn^4+^ by a redox shuttling agent of GSH/GSSG. **a** Schematic diagram of regenerable antioxidant mechanism of GSH in Pb–Sn perovskite. **b** Absorption spectra of GSH and GSH with Sn^4+^ in DTNB solution. **c** Absorption spectra of the reaction product of GSH and Sn^4+^ in DTNB solution, and after subsequent addition of Sn powder or Pb powder. **d** ^1^H NMR spectra of GSH, GSSG, and GSH with Sn^4+^. **e** Photographs depicting the color evolution of SnI_2_ solution with or without GSH exposed to ambient air. **f** XPS spectra of Pb–Sn perovskite films with or without GSH. **g** Steady-state PL evolution of Pb–Sn perovskite films with and without GSH with different exposure time in ambient air. **h** FTIR spectra of GSH and perovskite with GSH; **i** TOF–SIMS depth profile of Pb–Sn perovskite film with GSH

During device fabrication and operation, the Sn^2+^ ions are readily oxidized to Sn^4+^, and may also undergo disproportionation reactions yielding Sn^4+^ and metallic Sn^0^. Furthermore, metallic Pb^0^ forms through photodecomposition of residual PbI_2_ [30]. After incorporating the GSH-GSSG redox shuttle into perovskite film, Sn^4+^ can be reduced back to Sn^2+^ and Pb^0^ and Sn^0^ can be oxidized into Pb^2+^ and Sn^2+^, reversing the degradation processes as illustrated in Fig. 1a. To elucidate the redox reactions between perovskite and GSH (Fig. S2), we employed a quantitative detection method [31] to measure the concentration of free thiol groups based on the 5,5'-dithiobis(2-nitrobenzoic acid) (DTNB) assay. The detection mechanism (depicted in Fig. S3) involves the reaction of free thiols (R-SH) with DTNB^2−^ to generate 2-nitro-5-thiobenzoate anion (TNB^2−^), which exhibits characteristic absorption peak at 412 nm. This signature absorption enables precise quantification of the concentration of thiol compounds [32]. As shown in Fig. 1b, GSH exhibits a strong absorption peak at 412 nm in the presence of DTNB, attributed to its free thiol groups. However, upon the addition of Sn^4+^, the absorption intensity at 412 nm decreases and eventually disappears, indicating the oxidation of GSH to GSSG by Sn^4+^. Further evidence of the redox reaction between GSH and Sn^4+^ is provided by the rapid color change of SnI_4_ solution from orange red to bright yellow upon addition of GSH, as shown in Fig. S4. This color transition is consistent with previous observations of SnI_4_ being reduced to SnI_2_ [33], corroborating the results shown in Fig. S2a.

DTNB further demonstrated the reduction of GSSG upon adding metallic Sn or Pb powder. As illustrated in Fig. 1c, the enhanced absorption intensity at 412 nm signifies the regeneration of free thiol group resulting from the reduction of GSSG by either Sn or Pb powder. It is worth noting that the absorption intensity is stronger after adding Sn powder than Pb powder. In addition, similar absorption changes are observed when commercial GSSG was treated with Sn/Pb powders (Fig. S5), confirming that the product of reaction between GSSG and metallic Sn^0^ or Pb^0^ is GSH. These results elucidate a complete redox cycle: GSH is initially oxidized by Sn^4+^ to form GSSG, followed by the subsequent reduction of GSSG back to GSH by Sn/Pb powders.

To further elucidate the redox cycle at the molecular level, we employed ^1^H nuclear magnetic resonance (NMR) spectroscopy to characterize the reaction products. As shown in Fig. 1d, the *β*-carbon hydrogen of cysteine (Cys-*β*CH) in GSH exhibits a characteristic singlet at 2.97 ppm [34]. In commercial GSSG, this signal splits into two distinct peaks at 3.0 and 3.3 ppm, respectively, due to the oxidation-induced changes in chemical environment [35]. Upon the addition of Sn^4+^ into GSH, two split peaks emerge at 2.98 and 3.28 ppm, indicating formation of GSSG by Sn^4+^ oxidation. The slight shift in these peaks can be attributed to the coordination between the GSSG and Sn^4+^/Sn^2+^ ions [36]. The reduction of GSSG by Sn/Pb was also characterized by ^1^H NMR spectroscopy (Fig. S6). The characteristic peak of Cys-αCH in GSH is located at 4.59 ppm, which disappears in the GSSG [35]. However, when GSSG is treated with Sn/Pb powders, the characteristic peak of GSH at 4.59 ppm recovers, indicating the reduction of GSSG back to GSH by Sn/Pb. Additionally, the peak intensity for GSSG treated with Sn powder is significantly higher than that with Pb powder, showing stronger reducing ability of Sn compared to Pb, which is consistent with the results shown in Fig. S5. DTNB and NMR results confirm the proposed redox shuttle mechanism, revealing that GSH can eliminate harmful impurities in PSCs through a redox cycle analogous to its working mechanism in biological system.

The oxidation of Sn^2+^ to Sn^4+^ introduces electronic trap states in the film, severely degrading the optoelectronic properties of perovskite [37]. To address this issue, we investigated the effect of GSH incorporation on suppressing Sn^2+^ oxidation. As shown in Fig. 1e, the addition of GSH into SnI_2_ solution induces a distinct color change from orange yellow to bright yellow, attributed to the coordination of the carbonyl and carboxyl functional groups in the GSH and Sn^2+^ [38]. Notably, even after 120 min of air exposure, the SnI_2_ solution with GSH remains bright yellow, whereas the SnI_2_ solution without GSH turns dark red due to Sn^2+^ oxidation to Sn^4+^, demonstrating the excellent antioxidation properties of GSH. To further examine the antioxidation effect of GSH in Pb–Sn mixed perovskite films, X-ray photoelectron spectroscopy (XPS) was performed on perovskite films with GSH (Target) and without GSH (Control). As shown in Fig. 1f and Table S1, the Sn^4+^ content in the film is dramatically reduced from 13.12% in the control film to only 2.83% in the target film. These findings demonstrate that GSH effectively suppresses the Sn^2+^ oxidation in perovskite films, which is beneficial for enhancing the electrical properties and stability of perovskite films.

Conventional antioxidants or reducing agents in Pb–Sn perovskite primarily target Sn^4+^ but fail to address the disproportional products of Sn^2+^, which contains both Sn^4+^ and Sn^0^/Pb^0^. Furthermore, the continuous consumption of the antioxidation additives gradually diminishes their functionality. In contrast, the incorporation of GSH redox shuttle not only can remove the Sn^4+^, Sn^0^, and Pb^0^ impurities but also maintain its reducing capability through self-regeneration. To verify the impact of GSH on the Sn^0^/Pb^0^ metallic impurities, we conducted XPS measurements on aged perovskite films without GSH (Control) and with GSH (Target) after ambient air exposure. As shown in Fig. S7 and Table S2, substantial Sn^0^ and Pb^0^ were detected in the aged control film, whereas their concentrations were significantly reduced in the target film. These results confirm the removal of Sn^0^ and Pb^0^ by the GSH redox shuttle, providing strong evidence for the proposed redox mechanism. The time-dependent photoluminescence spectroscopy (PL) of perovskite film in ambient air further corroborated the enhanced antioxidation properties of GSH (Fig. 1g). After 60 min of air exposure, the target film with GSH still retained 74% of their initial PL intensity, compared to only 28% retention of the control film without GSH. In summary, the self-regenerating GSH effectively eliminates both oxidized (Sn^4+^) and reduced (Sn^0^ and Pb^0^) impurities, establishing a sustained protection against oxidation and degradation of Pb–Sn mixed perovskite.

To elucidate the interactions between GSH and Pb–Sn mixed perovskite, we performed Fourier transform infrared spectroscopy (FTIR) analysis. As shown in Fig. 1h, the incorporation of GSH into perovskite film results in the disappearance of the characteristic thiol (S–H) absorption peak at 2524 cm^−1^, indicating the oxidation of thiol groups to disulfide bonds [39]. This observation is consistent with the results in Fig. 1b, d, where the thiol group disappears as GSH is oxidized to GSSG by Sn^4+^ ions. Additionally, the carboxyl (1712 cm^−1^) and carbonyl (1660 cm^−1^) peaks in GSH both shift to lower wavenumbers, mainly attributed to the coordination between the carboxyl/carbonyl groups and Sn^2+^/Pb^2+^ [40]. The high-resolution S 2*p* XPS spectra (Fig. S8) reveal a characteristic disulfide signal at 164.02 eV in perovskite with GSH [41], further confirming the GSSG formation by the GSH oxidation. To study the distribution of GSH at different depths of the perovskite films, we conducted time of flight secondary ion mass spectrometry (TOF–SIMS) analysis on GSH incorporated perovskite. As shown in Fig. 1i, the depth profile of GSH, tracked by the S^−^ ion signal, reveals that GSH is predominantly distributed at the bottom and top surfaces of the perovskite film, particularly at the bottom surface. In addition, the (S–S)^−^ ion signal associated with disulfide groups of GSSG is also detected [42], corroborating the oxidation of GSH. This signal exhibits a similar interfacial distribution trend at the top and bottom surfaces, indicating the formation of a protective antioxidation shell at the perovskite film surface.

### Regulation of Crystallization Kinetics by GSH

**Fig. 2:**
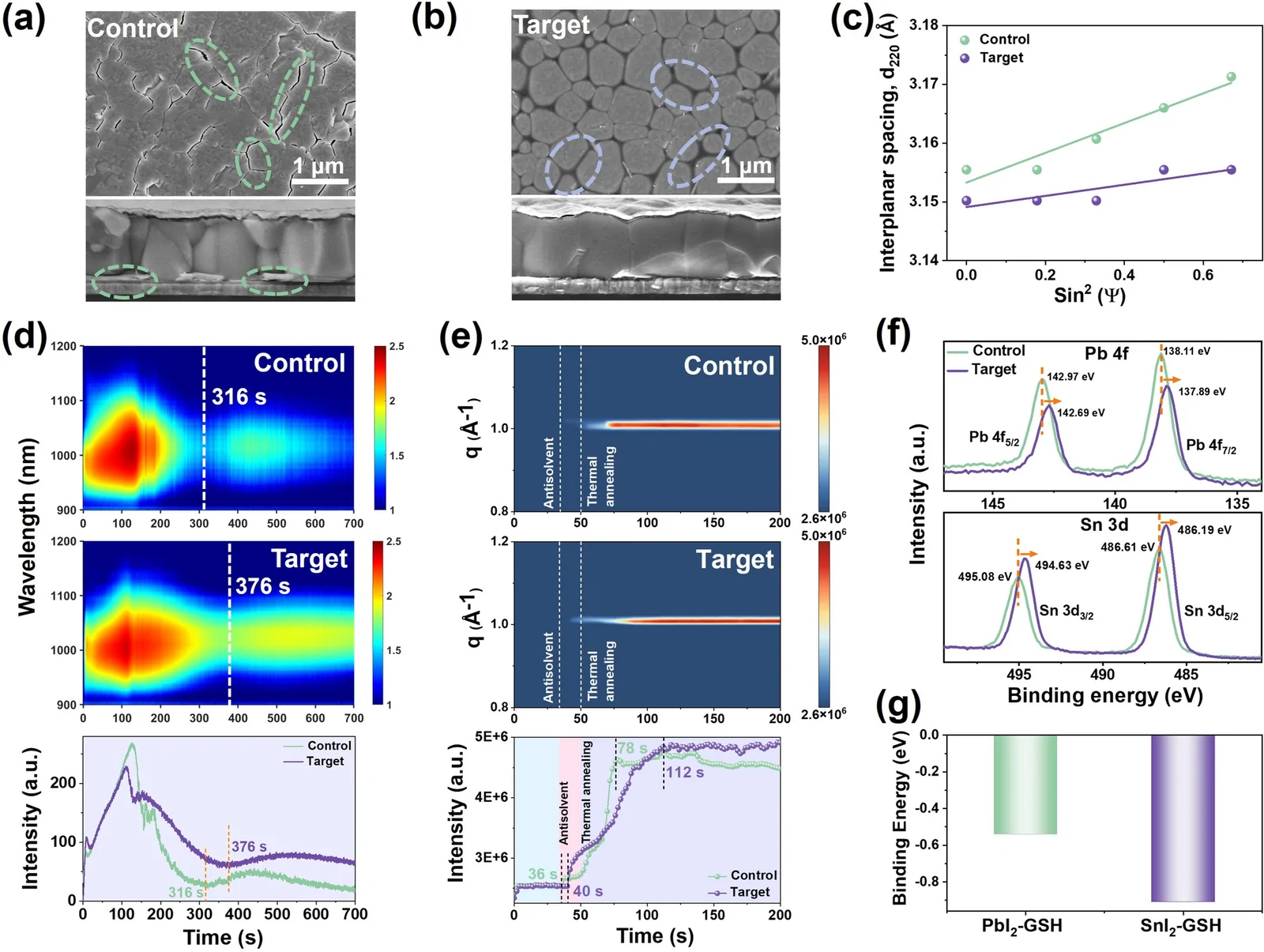
SEM images of the buried interface and cross section of the perovskite film **a** without GSH and **b** with GSH. **c** Interplanar spacing d_220_ versus sin^2^Ψ plots for the perovskite film with and without GSH, extracted from the GIXRD patterns. **d** Evolution of the PL spectra (upper panel) and corresponding PL intensity at 1017 nm (lower panel) for the perovskite with and without GSH during annealing process. **e** In situ GIWAXS pattern (upper panel) and the integrated peak intensities (lower panel) for Pb–Sn mixed perovskite films with and without GSH during fabrication process. (Antisolvent was applied at t = 34 s, followed by thermal annealing at t = 50 s.) **f** XPS spectra of Pb 4f and Sn 3d core levels of Pb–Sn mixed perovskite films with and without GSH. **g** Calculated binding energies of PbI_2_-GSH and SnI_2_-GSH complexes from DFT calculations

The incorporation of GSH redox shuttle into perovskite not only removes the redox impurities, but also suppresses defects in the perovskite films through the strong chemical interactions between GSH functional groups and perovskite. The morphology of perovskite films with and without GSH was characterized by scanning electron microscopy (SEM). The top-view SEM images (Fig. S9) demonstrate that the incorporation of GSH preserves the grain morphology of the perovskite film. Figure 2a and b presents the SEM images of the buried interface and cross section of perovskite films with and without GSH, respectively. The buried interface SEM image reveals numerous cracks at the grain boundaries of the control film (green circles), suggesting weak cohesion between grains [43]. In contrast, the target film exhibits densely packed grains with adhesive-like materials at the grain boundaries (blue circles). Energy-dispersive spectroscopy (EDS) characterization was performed to investigate the composition of these substances at the grain boundaries. As shown in Fig. S10, the grain boundary (site 1) contains 0.62% S and 4.46% O atoms (originating from GSH), while no S and only minimal O are detected within the grain interior (site 2). This observation indicates that GSH is primarily distributed at the grain boundaries in the perovskite film. The cross-sectional SEM image of control sample shows predominately small and incoherent grains, whereas the target film exhibits large columnar grains with improved crystallinity. Furthermore, compared to the control film, the target film exhibits better contact with the underlying hole transport layer (HTL), facilitating efficient carrier extraction. To further study the crystal structure and phase composition of the perovskite film, X-ray diffraction (XRD) analysis was performed. As shown in Fig. S11, the introduction of the GSH does not alter the crystal structure of the perovskite film. However, the intensity ratio of (100)/(110) peaks in the target film with GSH is much higher than that of the control film, indicating that the GSH promotes preferential orientation of perovskite crystals along the (100) plane, thereby enhancing crystal quality [44].

We subsequently employed grazing incident X-ray diffraction (GIXRD) to analyze the residual stress in perovskite films. Figure S12a, b shows the GIXRD patterns of the perovskite (220) crystallographic plane for control and target film, obtained at different depths with varying tilt angle Ψ. For the control film, the diffraction peak of (220) plane shifts to smaller angles as Ψ increased from 0° to 55°, corresponding to a monotonic increase in the interplanar spacing d_220_. In contrast, the GSH-modified perovskite film exhibits minimal variation in interplanar spacing d_220_. The interplanar spacing (d_220_) is plotted as a function of sin^2^Ψ, with linear fitting of the slope, as shown in Fig. 2c. The large positive slope observed in the control film indicates substantial residual tensile stress (92.61 MPa, as shown in Fig. S12c) primarily attributed to the large mismatch of the coefficient of thermal expansion (CTE) between the perovskite and substrate [45]. Notably, the perovskite film with GSH exhibited a significantly reduced slope with residual tensile stress of 34.77 MPa, suggesting effective tensile stress relief by GSH. Higher residual stress would cause severe lattice distortion in the perovskite, resulting in increased defects [46]. The smaller residual stress of GSH incorporated films can effectively alleviate stress induced defects, potentially contributing to enhanced device performance [47].

To investigate the influence of GSH redox shuttle on the kinetics of perovskite film crystallization, in situ UV–Vis absorption, PL, and synchrotron X-ray diffraction spectroscopy were employed to monitor the crystallization kinetics. Figure S13 presents the in situ UV–Vis absorption spectra (upper panel) and time-dependent evolution of absorption intensity at 700 nm (lower panel) for perovskite films with and without GSH during spin-coating process. Initially, the perovskite precursor solution shows negligible absorption, indicating the absence of perovskite nucleation until the introduction of gas quenching at t = 30 s. The perovskite film without GSH exhibits a distinctive absorption onset at 32.2 s, whereas the film with GSH demonstrated significant absorption later at 33.9 s. This delayed nucleation and crystallization in the presence of GSH can be attributed to the strong chemical interactions between GSH and perovskite constituents [48]. Moreover, in situ PL spectra (Fig. S14 upper panel) and the PL intensity at 958 nm (Fig. S14 lower panel) demonstrate a similar nucleation delay (from 35.9 to 37.7 s) upon GSH incorporation, corroborating the absorption spectroscopy results. Figure 2d illustrates the in situ PL spectra (upper panel) and PL intensity evolution at 1017 nm (lower panel) of perovskite films during annealing process. The PL intensity exhibits a sharp increase due to the rapid perovskite crystallization with solvent removal, followed by a dissolution recrystallization process during extended annealing (marked by the white line) [49]. Notably, the incorporation of the GSH significantly extends the onset time of the solvent recrystallization from 316 to 376 s, demonstrating its impact on modulating perovskite crystallization kinetics.

Figure 2e shows the time-resolved GIWAXS profiles (upper panel) and integrated peak intensities (lower panel) of the Pb–Sn perovskite films with and without GSH. For the perovskite precursor without GSH, the characteristic diffraction peak emerged at 36 s, indicating the onset of nucleation [50]. In contrast, the perovskite with GSH exhibited diffraction intensity onset at 40 s, confirming delayed nucleation. During thermal annealing with solvent removal, the intermediate solvent complex underwent progressive transformation into crystalline perovskite [51]. The incorporation of GSH extended the complete crystallization transition period from 78 to 112 s. These findings corroborate with the observations from in situ UV–Vis absorption and PL spectroscopies. The delayed crystallization process facilitates efficient ion diffusion and complete chemical reactions, thereby promoting the formation of high-quality perovskite films.

XPS analysis was performed to reveal the underlying mechanism of delayed crystallization. The XPS spectra (Fig. 2f) demonstrated that the GSH incorporation induced obvious shifts toward lower binding energies in both Pb 4*f* and Sn 3*d* peaks, primarily attributed to the strong coordination interactions between Pb^2+^/Sn^2+^ cations and the carbonyl/carboxyl functional groups of GSH [52]. Density functional theory (DFT) calculations further validated these strong chemical interactions, demonstrating binding energies of -0.54 and -0.91 eV for GSH-PbI_2_ and GSH-SnI_2_ complexes, respectively (Figs. 2g and S15). These findings suggest that the strong chemical interactions between GSH and PbI_2_/SnI_2_ modulate the crystallization process, resulting in delayed crystallization during spin-coating and extended solvent dissolution during thermal annealing.

### Optoelectronic Properties of Pb–Sn Mixed Perovskites with GSH

**Fig. 3:**
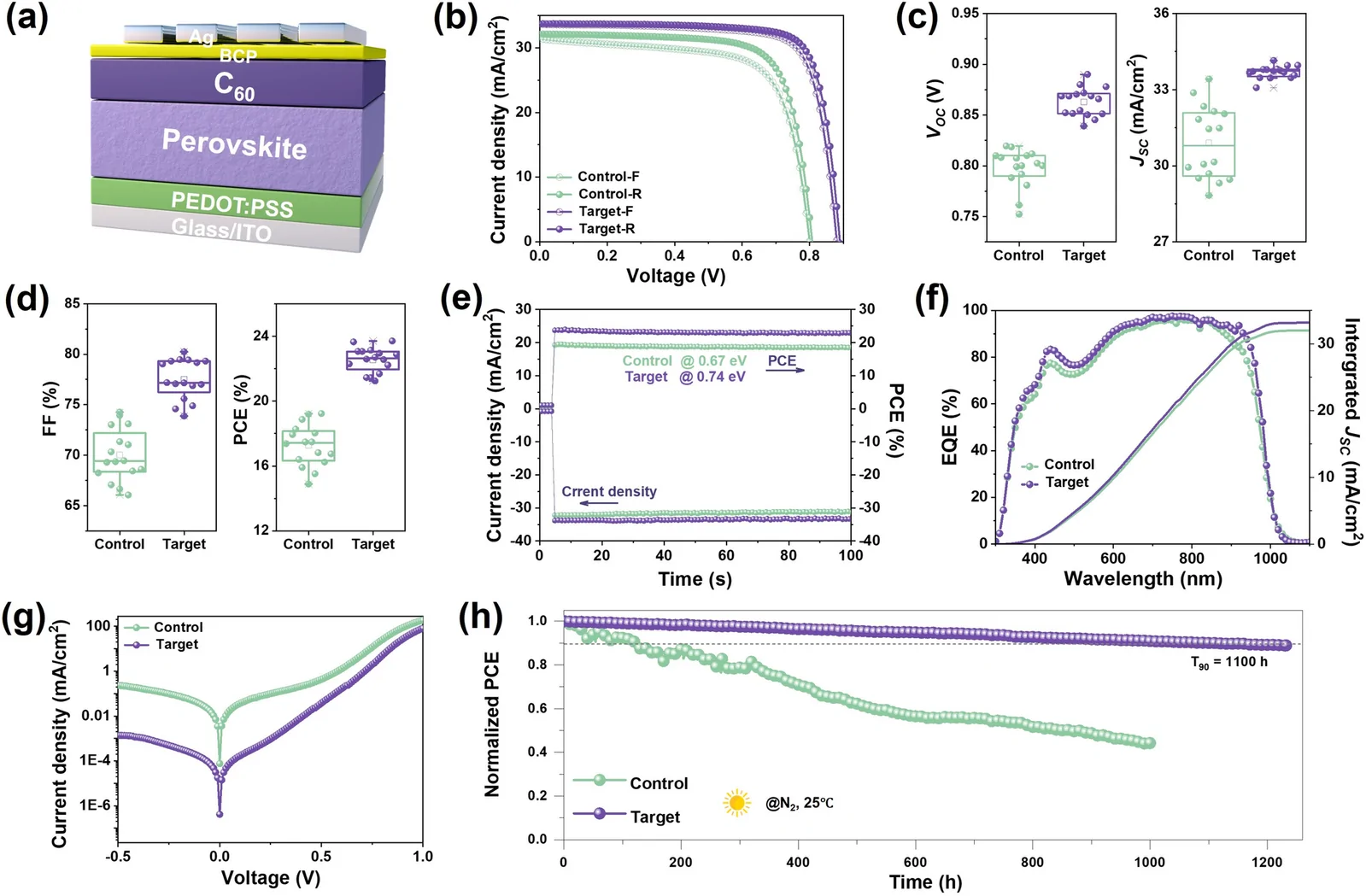
**a** Schematic diagram of the device structure for Pb–Sn mixed PSCs. **b** *J-V* curves of champion PSCs with and without GSH. Statistical distribution of photovoltaic parameters of **c** *V*_*OC*_ and *J*_*SC*_, and **d** FF and PCE for the Pb–Sn mixed PSCs with and without GSH. **e** Steady-state power output measured at maximum power point, **f** EQE spectra and **g** dark *J-V* curves of PSCs with and without GSH. **h** PCE decay of unencapsulated PSCs with and without GSH under continuous illumination in a nitrogen-filled glove box

Motivated by the excellent impurity removal efficacy and enhanced perovskite crystallinity, we investigated the impact of GSH incorporation on the device performance. We fabricated inverted *p-i-n* planar PSCs with the structure of indium tin oxide (ITO)/poly(3,4-ethylenedioxythiophene) polystyrene sulfonate (PEDOT:PSS)/FA_0.7_MA_0.3_Pb_0.5_Sn_0.5_I_3_/C_60_/bathocuproine (BCP)/Ag, incorporating 1 mol% GSH (relative to SnI_2_) in the precursor solution, as shown in Fig. 3a. Figure 3b presents the current density–voltage (*J-V*) curves of champion PSCs with and without GSH, with the corresponding photovoltaic parameters summarized in Table S3. The GSH-modified PSCs exhibited markedly improved photovoltaic parameters across all metrics: open-circuit voltage (*V*_*OC*_) increased from 0.81 to 0.89 V, short-circuit current density (*J*_*SC*_) from 32.14 to 33.72 mA cm^−2^, and fill factor (FF) from 73.95 to 79.03%, yielding an impressive PCE enhancement from 19.19 to 23.71%. Notably, the hysteresis index decreased from 8.91 to 3.50%, attributed to the reduced defect density and the enhanced film quality by GSH incorporation [53]. Figure 3c, d shows the statistical distributions of photovoltaic parameters (*V*_*OC*_, *J*_*SC*_, FF, and PCE) of the PSCs with and without GSH (16 devices per configuration). It reveals that the photovoltaic performance enhancement with GSH incorporation primarily stems from improved *V*_*OC*_ and FF, suggesting reduced non-radiative recombination and enhanced carrier extraction. Figure 3e presents the stabilized power output (SPO) of devices recorded at their maximum power point (MPP). The GSH-modified PSC exhibited superior steady-state PCE of 23.13% with a corresponding current density of 33.39 mA cm^−2^, significantly outperforming the control device (stabilized PCE of 18.64% and current density of 31.28 mA cm^−2^). External quantum efficiency (EQE) measurements yielded integrated current densities of 31.99 and 33.16 mA cm^−2^ for devices without and with GSH, respectively (Fig. 3f), validating the *J*_*SC*_ values obtained from the *J-V* curves [54]. Furthermore, the dark current density of the GSH-modified PSC is two orders of magnitude lower than that of the control device (as shown in Fig. 3g), indicating that GSH significantly reduces leakage current. This improvement can be attributed to lower defect density and fewer pinholes at the buried interface.

In addition, the long-term operational stability of PSCs with and without GSH was evaluated in a N_2_ atmosphere under continuous illumination. As shown in Fig. 3h, the GSH-modified PSC retained 90.86% of its initial PCE after 1000 h of continuous 1 sun illumination, whereas the PCE of the control device decreased to 44% of its initial value under identical conditions. These results demonstrate that the GSH incorporation significantly enhances the stability of PSCs, owing to the self-elimination of redox impurities during device operation.

**Fig. 4:**
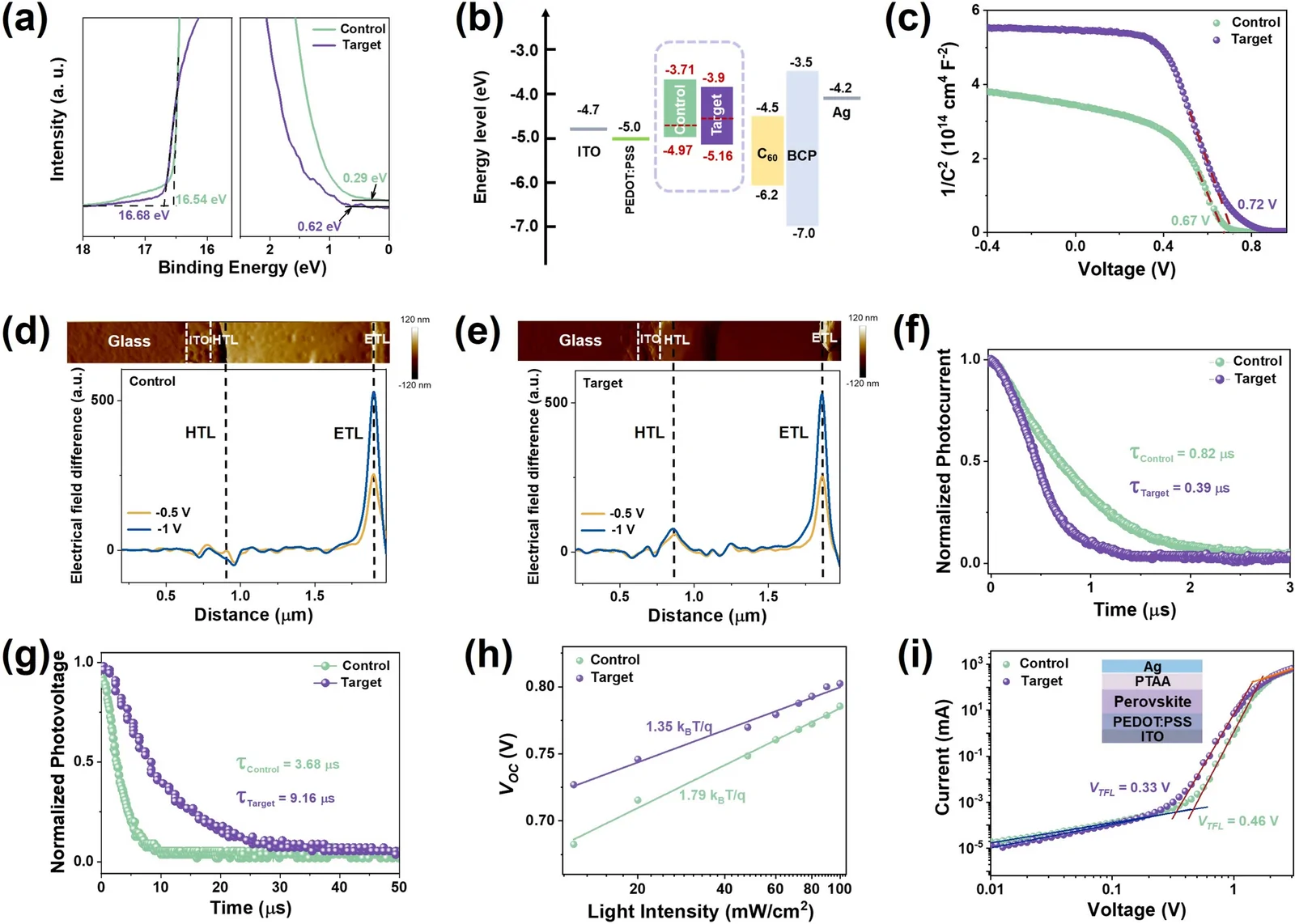
**a** Secondary electron cutoff energies and onsets of the *E*_V_ from UPS spectra for Pb–Sn mixed perovskite with and without GSH. **b** Energy band alignment of the perovskite films with or without GSH, along with other adjacent layers in PSCs. **c** Mott–Schottky plots for PSCs with and without GSH. Cross-sectional electric field distribution of PSCs **d** without GSH and **e** with GSH measured by KPFM under varying bias voltages. **f** TPC decay curves and **g** TPV decay curves of PSCs with or without GSH. **h** *V*_*OC*_ versus light intensity of Pb–Sn mixed PSCs with and without GSH. **i** Space charge limited current (SCLC) spectra of hole-only devices

We subsequently conducted comprehensive characterization of perovskite film and devices to elucidate the mechanism underlying PSC performance improvement. UV–vis absorption and ultraviolet photoelectron spectroscopy (UPS) characterization were performed to analyze the energy levels of the perovskite films. The secondary electron cutoff energy (*E*_cutoff_) and the onsets of the valence band maximum (*E*_V_) were determined from the UPS spectra, as shown in Fig. 4a. In addition, Tauc plot analysis (Fig. S16) revealed identical band gaps (*E*_g_) of 1.26 eV for perovskite films with and without GSH. The GSH incorporation preserves the intrinsic band gap of perovskite. The energy level results are summarized in Table S4 and the energy band alignment of perovskite films with adjacent functional layers in the PSCs are depicted in Fig. 4b. It is observed that both the conduction band minimum (*E*_C_) and *E*_V_ of perovskite film exhibited a downward shift of 0.19 eV after GSH incorporation. Therefore, the GSH incorporation induces favorable band bending at the top surface of perovskite, which promotes the extraction of electrons from the perovskite to the electron transport layer and increases the barrier of hole back flowing [55]. Notably, the Fermi level (*E*_f_) of the GSH-modified perovskite film shifts upward by 0.14 eV, showing that the GSH incorporation mitigates the p-type self-doping of the perovskite film due to oxidation. This improvement is primarily attributed to the regenerable antioxidant mechanism of the GSH redox shuttle [56, 57]. Mott–Schottky (M-S) analysis was performed to investigate the built-in potential (*V*_bi_) of the devices. As shown in Fig. 4c, GSH-incorporated device exhibited an enhanced *V*_bi_ of 0.72 V compared to 0.67 V for the control device, indicating stronger driving force for carrier extraction and separation in the GSH-incorporated devices [58]. In addition, the hole carrier concentration extracted from the slope of the M-S curve is reduced from 3.02 × 10^15^ to 2.48 × 10^15^ cm^−3^ upon GSH incorporation (Table S5), confirming the alleviated p-type self-doping of the perovskite due to Sn^2+^ oxidant through GSH redox shuttle.

To further investigate the charge carrier transport and separation at the perovskite interface, we measured surface potential distributions across device cross section using Kelvin probe force microscopy (KPFM). Figure S17a, b shows the surface potential distribution of the devices with and without GSH at bias voltages of -1 and -0.5 V, respectively. To eliminate the influence of static surface charge, the 0 V voltage profile is subtracted from each bias measurement to obtain potential difference curves (Fig. S17c, d). The electric field difference was subsequently calculated through the first-order derivative of the potential difference (Fig. 4d, e). It is observed that both the devices with and without GSH exhibited prominent electric field peaks at the perovskite/ETL interface, suggesting effective charge-separating junction at the ETL interface [59]. However, the control device exhibited negligible field strength at the HTL/perovskite interface, which is due to the severe shunting caused by the leakage junction at the HTL interface [60]. In contrast, the device with GSH exhibited a strong electric field peak at the HTL/perovskite interface, demonstrating enhanced junction quality [60]. This junction improvement is attributed to preferential GSH accumulation at perovskite buried interface, as evidenced by the morphology analysis (Figs. 1i, 2b). The resultant enhanced junction quality facilitates efficient carrier separation and extraction, fundamentally explaining the significant enhancement in *V*_*OC*_ and FF observed in GSH-incorporated PSCs.

Carrier dynamics in PSCs were further examined by transient photocurrent (TPC) and transient photovoltage (TPV) measurements. As shown in Fig. 4f, TPC analysis revealed decreased photocurrent lifetime from 0.82 to 0.39 μs in GSH-incorporated device, indicating enhanced carrier extraction [61, 62]. The enhancement of carrier extraction mainly stems from the improved junction quality at HTL/perovskite interface and optimized energy level alignment at the perovskite/ETL interface. Furthermore, TPV analysis (Fig. 4g) demonstrated extended carrier lifetime from 3.68 to 9.16 μs upon GSH incorporation, suggesting effective defect passivation and reduced non-radiative recombination rate [63]. Steady-state photoluminescence (PL) measurements were conducted to further investigate the carrier recombination. Figure S18 illustrates the PL spectra of the control and target films, with excitation from buried interface and top surface of the perovskite films, respectively. Enhanced PL intensity was observed for GSH-incorporated perovskite films at both sides, indicating significantly suppressed non-radiative recombination at both the buried and top interfaces [64].

To further explore the suppression of non-radiative recombination, the light-intensity-dependent *V*_*OC*_ measurements were performed, as shown in Fig. 4h. The ideality factor n decreased significantly from 1.79 for the control device to 1.35 for the GSH-incorporated devices. This result suggests that the defect-assisted non-radiative recombination is effectively suppressed, consistent with observed improvements in *V*_*OC*_ and FF [65]. To quantify the defect density, space-charge limited current (SCLC) measurement was conducted on hole-only device with the structure of ITO/PEDOT:PSS/perovskite/PTAA/Ag. As shown in Fig. 4i, the trap filling limit voltage (*V*_TFL_), determined from the transition point between ohmic and trap-filled limited (TFL) regions [66], decreased from 0.46 to 0.33 V upon GSH incorporation. Correspondingly, the calculated hole trap density (*N*_t_) (Table S6) decreased from 3.26 × 10^15^ to 2.33 × 10^15^ cm^−3^, attributed to suppressed Sn^2+^ oxidation and disproportion reactions and enhanced perovskite film crystallinity by GSH incorporation.

### Photovoltaic Performance of the All-Perovskite TSCs

**Fig. 5:**
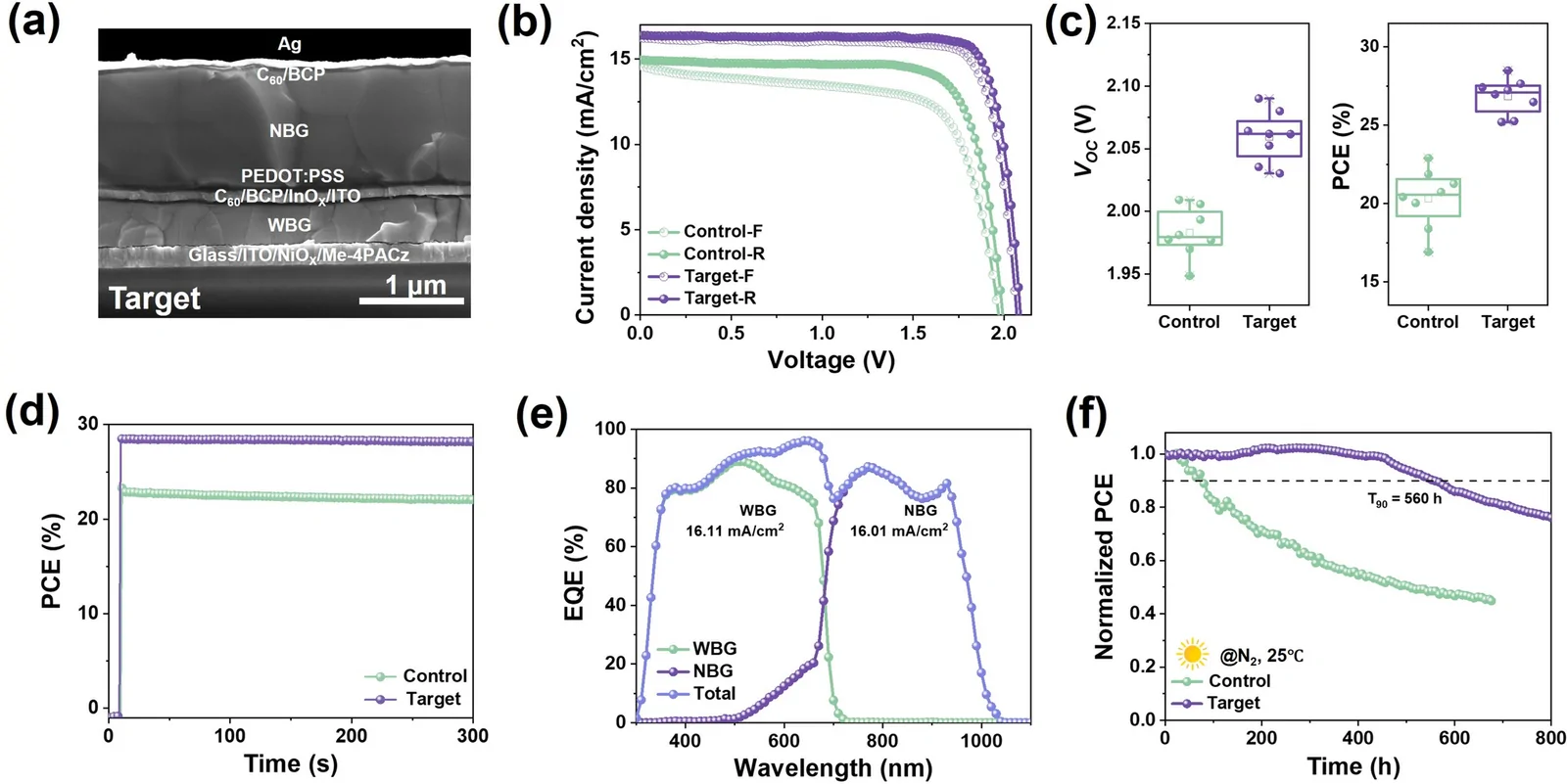
All-perovskite two-terminal (2T) tandem solar cells (TSCs). **a** Cross-sectional SEM image of 2T all-perovskite TSCs with GSH. **b** *J-V* curves of champion all-perovskite TSCs without and with GSH. **c** Statistical distribution of *V*_*OC*_ and PCE for all-perovskite TSCs with and without GSH. **d** Steady-state output at the maximum power point and **e** EQE spectra for all-perovskite TSCs with GSH. **f** PCE decay of unencapsulated all-perovskite TSCs with and without GSH under continuous illumination in a nitrogen-filled glove box

The superior PCE performance of Pb–Sn mixed PSC with GSH incorporation enables the fabrication of high-performance tandem cells. We subsequently fabricated a two-terminal (2T) all-perovskite tandem cells with the structure of ITO/NiO_X_/Me-4PACz/wide-bandgap (WBG) perovskite (FA_0.8_Cs_0.2_PbI_1.8_Br_1.2_)/C_60_/BCP/InO_X_/ITO/PEDOT:PSS/narrow-bandgap (NBG) perovskite (FA_0.7_MA_0.3_Pb_0.5_Sn_0.5_I_3_)/C_60_/BCP/Ag. Figures 5a and S19 illustrate cross-sectional SEM images of the tandem cells with GSH (target) and without GSH (control), respectively. It is evident that GSH incorporation promoted the formation of large columnar grains in NBG perovskite, contrasting with fine grains observed in the control devices. The bandgap of WBG perovskite is preciously tuned to 1.79 eV to fulfill the current matching requirement in tandem cells [67, 68]. The composition and fabrication procedure of WBD PSC is adopted from our previous work [69]. Figure S20 shows the *J-V* curves of the WBG PSCs, with performance parameters summarized in Table S7. The WBG PSC exhibits a *V*_*OC*_ of 1.33 V, a *J*_*SC*_ of 18.10 mA cm^−2^, an FF of 84.20%, and a PCE of 20.27%. The external quantum efficiency (EQE) measurements show that the integrated current density of the WBG PSC is 17.47 mA cm^−2^ (Fig. S21a), verifying the reliability of the *J-V* curve. The steady-state power output (SPO) (shown in Fig. S21b) under the MPP further confirmed the excellent performance of the WBG PSC, with a steady-state PCE of 19.96%.

Figure 5b presents the *J-V* curves of the champion tandem PSCs with and without GSH. The device with GSH achieved a *V*_*OC*_ of 2.09 V, a *J*_*SC*_ of 16.40 mA cm^−2^, and an FF of 83.15%, yielding an impressive PCE of 28.49% (Table S8). Moreover, the hysteresis index decreased from 13.89 to 4.14%, attributed to the enhanced NBG perovskite film quality. The statistics of photovoltaic parameter in Figs. 5c and S22 demonstrate excellent reproducibility of the GSH-incorporated tandem device. In addition, the performance improvement mainly stems from enhanced *V*_*OC*_ and FF. Figure 5d shows that the SPO of the GSH-incorporated tandem device under MPP is 28.18%, with negligible degradation during the measurement, whereas the control device exhibits a SPO of 22.06%. As shown in Fig. 5e, the integrated *J*_*SC*_ obtained from the EQE of the WBG and NBG subcells is 16.11 and 16.01 mA cm^−2^, respectively, demonstrating excellent current matching in the tandem device. The long-term operational stability of the tandem PSCs was further investigated under continuous 1 sun illumination in N_2_ atmosphere (Fig. 5f). The GSH-incorporated tandem device retained 90% of the initial PCE after 560 h, whereas the PCE of the control device decreased to 48% of its initial value. These results highlight the significant role of the GSH redox shuttle in enhancing the stability of all-perovskite tandem solar cells.

## Conclusion

In conclusion, we have introduced a nature-inspired redox shuttle GSH with regenerable antioxidant properties and crystallization modulation capabilities for Pb–Sn mixed PSC. The incorporation of GSH redox shuttle can effectively eliminate the harmful Sn^4+^ and Sn^0^/Pb^0^ redox impurities through the reversible GSH-GSSG cycles. This regenerable antioxidation mechanism reduces the hole defect density and improves solar cell stability. The strong chemical interactions between GSH and perovskite modulate crystallization process by regulating the nucleation and growth kinetics, significantly enhancing the perovskite film quality. GSH incorporation reduces the residual tensile stress at perovskite buried interface and suppresses defect formation. GSH incorporation facilitates charge extraction and separation through adjusting energy level alignment at charge transport layer interfaces. As a result, the single-junction Pb–Sn PSC achieves an impressive PCE of 23.71%, while the PCE of the champion all-perovskite TSC reaches 28.49%. Moreover, the GSH redox shuttle significantly enhances the device stability. The single-junction device retained 90.86% of the initial PCE after 1000 h of continuous illumination in N_2_ atmosphere. This work establishes a promising natural redox shuttle strategy for simultaneous antioxidation and crystallization modulation in Pb–Sn mixed PSC. These findings pave the way for the development of efficient and stable all-perovskite tandem solar cells.

## Supplementary Information

Below is the link to the electronic supplementary material.

Supplementary file1 (DOCX 7928 kb)
