# Supplementary material for: Nature-Inspired Redox Shuttle with Regenerable Antioxidant for Efficient All-Perovskite Tandem Solar Cells
Source: Nanomicro Lett. 2026 Jan 5;18:165. doi: 10.1007/s40820-025-02006-6 (PMC12765756; doi:10.1007/s40820-025-02006-6)
Supplement: Supplementary file 1 — Supplementary file1 (DOCX 7928 kb) [file 40820_2025_2006_MOESM1_ESM.docx]

# Supporting Information for

**Nature-Inspired Redox Shuttle with Regenerable Antioxidant for Efficient All-Perovskite Tandem Solar Cells**

Rui Meng^1,2†^, Liming Du^1,2†^, Can Li^1,2*^, Zhi Wan^1,2^, Jishan Shi^2^, Yueying Zhang^3^, Wenfeng Liu^2^, Chongyang Zhi^2^, Chunmei Jia^2^, Lili Tan^2^, Chuanxiao Xiao^3,4^, Xian-Zong Wang^5^, Lin Song^6^, Xingyu Gao^7^, Zhen Li^1,2*^

^1^ Shenzhen Research institute of Northwestern Polytechnical University, Sanhang Science & Technology Building, No.45th, Gaoxin South 9th Road, Nanshan District, Shenzhen City, 518057, P. R. China

^2^ State Key Laboratory of Solidification Processing, Center for Nano Energy Materials, School of Materials Science and Engineering, Northwestern Polytechnical University and Shaanxi Joint Laboratory of Graphene (NPU), Xi'an 710072, P. R. China

^3^ Ningbo Institute of Materials Technology and Engineering, Chinese Academy of Sciences, Ningbo 315201, P. R. China

^4^ Ningbo New Materials Testing and Evaluation Center CO., Ltd, Ningbo 315201, P. R. China

^5^ Center of Advanced Lubrication and Seal Materials, Northwestern Polytechnical University, Xi’an 710072, P. R. China

^6^ State Key Laboratory of Flexible Electronics (LOFE)&Institute of Flexible Electronics (IFE), Northwestern Polytechnical University, 127 West Youyi Road, Xi'an 710072, P. R. China

^7^ Shanghai Synchrotron Radiation Facility (SSRF), Zhangjiang Laboratory, Shanghai Advanced Research Institute, Chinese Academy of Sciences, 239 Zhangheng Road, Shanghai 201204, P. R. China

†Rui Meng and Liming Du contributed equally to this work.

*Corresponding authors. E-mail: [lican@nwpu.edu.cn](mailto:lican@nwpu.edu.cn) (Can Li); [lizhen@nwpu.edu.cn](mailto:lizhen@nwpu.edu.cn) (Zhen Li)

**S1 Detection of free thiol groups by DTNB assay**

Detection of free thiol groups by DTNB assay. The DTNB stock solution was prepared by dissolving 2 mg of DTNB in 0.5 mL of the Assay Buffer with thorough mixing. Aliquots were stored at -20℃ for future use, except for the portion used immediately. The DTNB working solution was obtained by mixing the stock solution with Assay Buffer at a volume ration of 1:35 (v/v) ratio. For each sample, 200 μL of the working sample was used. For the GSH sample, GSH was dissolved in deionized H_2_O to prepare a 0.1 mM solution, denoted as “Solution A”. For GSH with Sn^4+^, 0.1 mmol SnCl_4_·5H_2_O was added to solution A (denoted as “solution B”). For GSH with Sn^4+^ added with Sn/Pb, 0.1 mmol Sn powder was added to solution B (denoted as “solution C”) or 0.1 mmol Pb powder added to solution B (denoted as “solution D”). For GSSG, GSSG was dissolved in deionized H_2_O to prepare a 0.1 mM solution (denoted as “solution E”). For GSSG with Sn/Pb, 0.1 mmol Sn powder was added to solution E (denoted as “solution F”) or 0.1 mmol Pb powder added to solution E (denoted as “solution G”). 10 μL of each solution (A-G) was added dropwise to 200 μL of DTNB working solution, and the absorption spectra were recorded after standing for 5 min.

**S2 DFT calculation method**

Density functional theory as implemented in the Vienna Ab-initio Simulation Package (VASP) [S1, S2] was employed to optimize geometry structures. The exchange-correlation interactions were described by the generalized gradient approximation (GGA) in the form of the Perdew-Burke-Ernzerhof functional (PBE) [S3]. We have chosen the projected augmented wave (PAW) potentials [S4, S5] to describe the ionic cores and take valence electrons into account using a plane wave basis set with a kinetic energy cutoff of 500 eV. The electronic energy was considered self-consistent when the energy change was smaller than 10^-5^ eV. A geometry optimization was considered convergent when the force change was smaller than 0.04 eV/Å. The vacuum spacing in a direction perpendicular to the plane of the structure is 25 Å. The van der Waals interactions were considered by the method of the Grimme (DFT+D3) [S6]. The Brillouin-zone integration was sampled with a Monkhorst-Pack mesh of 2 × 2 × 1 in the structural relaxation calculations.

The binding energy (E_b_) is calculated by the Eq. (S1):

$E_{b}=E_{{Pb}/{{SnI}_{2}}-Glutathione}-E_{{Pb}/{{SnI}_{2}}}-E_{Glutathione}$ (S1)

where $E_{{Pb}/{{SnI}_{2}}-Glutathione}$ is the energy of total system, $E_{{Pb}/{{SnI}_{2}}}$ and $E_{Glutathione}$are the energy of Pb/SnI_2_ structure and Glutathione, respectively.

**Note S1 Space-Charge-Limited Current (SCLC) Analysis**

The trap density (*N*_trap_) is derived from the trap-filled limit voltage (*V*_TFL_) using the equation:

$N_{trap}=\frac{2\varepsilon_{r}\varepsilon_{0}V_{TFL}}{eL^{2}}$ (S2)

where *e* is the elementary charge, *N*_trap_ is the trap density, *L* is the thickness of the perovskite layer, *ε*_r_ is the relative dielectric constant, and *ε*_0_ is the vacuum permittivity. The hole trap density can be determined by identifying *V*_TFL_.

**Note S2 Mott-Schottky Analysis**

The Mott-Schottky relationship is given by:

$\frac{1}{C^{2}}=\frac{2}{e\varepsilon_{r}\varepsilon_{0}N}\left( V-V_{bi}-\frac{k_{B}T}{e} \right)$ (S3)

where *C* is the capacitance, *e* is the elementary charge, *ε*_r_ is the relative dielectric constant of the perovskite, *ε*_0_ is the vacuum permittivity, *N* is the charge carrier density, *V* is the applied bias voltage, *V*_bi_ is the built-in potential, *k*_B_ is the Boltzmann constant, and *T* is the temperature. The hole carrier concentration *N* can be calculated from the slope of the the linear region in *1/C^2^* vs. *V* plot.

**Supplementary Figures and Tables**

**Fig. S1** Schematic illustration of the GSH-GSSG redox shuttle mechanism in biological systems. Under oxidative conditions, the sulfhydryl groups from two GSH molecules form a disulfide bond, producing GSSG. The oxidized form, GSSG, is subsequently reduced back to GSH by glutathione reductase (GR), utilizing reduced nicotinamide adenine dinucleotide phosphate (NADPH) as an electron donor. Enzymes: GPx (glutathione peroxidase), GR (glutathione reductase). Cofactors: NADPH (reduced nicotinamide adenine dinucleotide phosphate), NADP^+^ (oxidized nicotinamide adenine dinucleotide phosphate)

**Fig. S2** The proposed redox reactions between perovskite and GSH. (**a**) Reduction of Sn^4+^ by GSH. (**b**) Regeneration of GSH from GSSG mediated by Sn^0^/Pb^0^

**Fig. S3** Schematic representation of reaction mechanism for DTNB-based quantitative detection of free thiol groups. Reaction pathway: Free thiol groups (R-SH) react with 5,5'-dithiobis-2-nitrobenzoic acid) (DTNB^2-^) via nucleophilic substitution, producing the chromogenic 2-nitro-5-thiobenzoate anion (TNB^2-^) and a mixed disulfide (R-S-TNB). Quantification principle: The TNB^2-^ anion exhibits a characteristic absorption peak at 412 nm, enabling accurate spectrophotometric quantification of thiol concentration.

**Fig. S4** Photographs of SnI_4_ solution before (left, orange-red) and after (right, bright yellow) GSH addition

**Fig. S5** Absorption spectra of commercial GSSG alone and following the addition of Sn powder or Pb powder in DTNB solution

**Fig. S6** ^1^H NMR spectra of GSSG, GSH, and GSSG after treatment with Sn powder or Pb powder

**Fig. S7** XPS spectra of aged Pb-Sn mixed perovskite films under ambient air exposure: (a) Sn 3d and (b) Pb 4f core levels without GSH; (c) Sn 3d and (d) Pb 4f core levels with GSH

**Fig. S8** S 2p core level XPS spectra of Pb-Sn mixed perovskite films (a) without GSH and (b) with GSH

**Fig. S9** Top-view SEM images of perovskite films (**a**) without GSH and (**b**) with GSH

**Fig. S10.** Energy dispersive spectrometer (EDS) analysis of Pb-Sn perovskite film with GSH: (**a**) SEM image, (**b**) atomic contents of S and O corresponding at sites 1 and 2

**Fig. S11** (**a**) XRD patterns of the perovskite film with and without GSH, (**b**) peak intensity ratios of the (100) plane and other planes in the XRD patterns shown in **Fig.** (a)

**Note S3** The residual stress *σ_R_* in the perovskite film is quantified using the following equation [S7]:

$\sigma_{R}=\left( \frac{E_{(220)}}{1+\upsilon} \right)\left( \frac{m}{d_{n}} \right)$ Equations (S4)

where *E_(220)_* is the Young's modulus of the perovskite film along the (220) direction; *υ* is the Poisson's ratio; *m* is the slope and *d_n_* is the y intercept of the linear fit in **Fig.** 2c [S8].

**Fig. S12** GIXRD patterns of Pb-Sn perovskite films (a) without and (b) with GSH at different Ψ angles (0° to 55°). (c) The calculated residual stress *σ_R_* of perovskite films with and without GSH

**Fig. S13** (**a**) The evolution of the in-situ UV-vis absorption spectra and (**b**) corresponding absorption intensity at 700 nm for perovskite films with and without GSH during spin-coating process

**Fig. S14** (**a**) The evolution of the PL spectra and (**b**) corresponding PL intensity at 958 nm for perovskite films with and without GSH during spin-coating process

**Fig. S15** Interaction diagrams of (**a**) PbI_2_-GSH and (**b**) SnI_2_-GSH complexes obtained from DFT calculations

**Fig. S16** (**a**) Absorbance spectra of the perovskite films with and without GSH. Tauc plots of the perovskite films (**b**) without GSH and (**c**) with GSH

**Fig. S17** Surface potential distribution on the cross-section of Pb-Sn PSCs (**a**) without GSH and (**b**) with GSH at different bias voltages, measured by KPFM. Potential difference curves on the cross section of Pb-Sn PSCs (**c**) without GSH and (**d**) with GSH

**Fig. S18** PL spectra excited from (**a**) buried interface and (**b**) top interface of perovskite films with and without GSH

**Fig. S19** Cross-sectional SEM images of all-perovskite TSCs without GSH

**Fig. S20** *J-V* curves of the wide-bandgap PSCs based on FA_0.8_Cs_0.2_PbI_1.8_Br_1.2_

**Fig. S21** (a) EQE spectra and (b) steady-state power output at maximum power point for the wide-bandgap PSCs based on FA_0.8_Cs_0.2_PbI_1.8_Br_1.2_

**Fig. S22.** Statistical distribution of *J_SC_* and FF for all-perovskite TSCs with and without GSH

**Table S1** The contents of Sn^2+^ and Sn^4+^ in the XPS spectra of Pb-Sn mixed perovskite films with and without GSH

|  | **Sn^2+^ (%)** | **Sn^4+^ (%)** |
| --- | --- | --- |
| **Control** | **86.88** | **13.12** |
| **Target** | **97.17** | **2.83** |

**Table S2** The contents of Sn^0^ and Pb^0^ in the XPS spectra measurement on aged perovskite films with GSH (Target) or without GSH (Control) under air exposure

|  | Sn^0^ (%) | Pb^0^ (%) |
| --- | --- | --- |
| Control | 6.39 | 17.67 |
| Target | 3.35 | 3.96 |

**Table S3** Photovoltaic parameters of Pb-Sn mixed PSCs with and without GSH

|  | *V_OC_*  (V) | *J_SC_* (mA/cm^2^) | FF  (%) | PCE  (%) | Hysteresis index (%) |
| --- | --- | --- | --- | --- | --- |
| Control-F | 0.80 | 31.45 | 69.46 | 17.48 | 8.91 |
| Control-R | 0.81 | 32.14 | 73.95 | 19.19 |  |
| Target-F | 0.88 | 33.67 | 77.19 | 22.88 | 3.50 |
| Target-R | 0.89 | 33.72 | 79.03 | 23.71 |  |

**Table S4** Summary of the energy level results of the perovskite films in PSCs with and without GSH

| Sample | E_g_ (eV) | E_C_ (eV) | E_V_ (eV) | E_f_ (eV) |
| --- | --- | --- | --- | --- |
| Control | 1.26 | -3.71 | -4.97 | -4.68 |
| Target | 1.26 | -3.90 | -5.16 | -4.54 |

**Table S5** The built-in potential and the hole carrier concentration of PSCs with or without GSH based on the Mott-Schottky (M-S)

| Sample | *V_bi_* (V) | *N*_t_*-H* (cm^-3^) |
| --- | --- | --- |
| Control | 0.67 | 3.02×10^15^ |
| Target | 0.72 | 2.48×10^15^ |

**Table S6** Trap density of PSCs with or without GSH derived from the space-charge-limited current (SCLC) method

| Sample | *V_TFL_* (V) | *N_t_* (cm^-3^) |
| --- | --- | --- |
| Control | 0.46 | 3.26×10^15^ |
| Target | 0.33 | 2.33×10^15^ |

**Table S7** Photovoltaic parameters of the wide-bandgap PSCs

|  | *V_OC_* (V) | | *J_SC_* (mA/cm^2^) | FF (%) | PCE (%) |
| --- | --- | --- | --- | --- | --- |
| WBG-F | | 1.33 | 18.20 | 81.20 | 19.65 |
| WBG-R | | 1.33 | 18.10 | 84.20 | 20.27 |

**Table S8** Photovoltaic parameters of all-perovskite TSCs with and without GSH

|  | *V_OC_* (V) | | *J_SC_* (mA/cm^2^) | FF (%) | PCE (%) | Hysteresis index (%) |
| --- | --- | --- | --- | --- | --- | --- |
| Control-F | | 1.97 | 14.59 | 68.57 | 19.72 | 13.89 |
| Control-R | | 1.99 | 14.96 | 76.79 | 22.90 |  |
| Target-F | | 2.07 | 16.25 | 81.17 | 27.31 | 4.14 |
| Target-R | | 2.09 | 16.40 | 83.15 | 28.49 |  |

**Supplementary References**

1. G. Kresse, J. Furthmüller, Efficiency of ab-initio total energy calculations for metals and semiconductors using a plane-wave basis set. Comput. Mater. Sci. **6**(1), 15–50 (1996). <https://doi.org/10.1016/0927-0256(96)00008-0>
2. G. Kresse, J. Furthmüller, Efficient iterative schemes for *ab initio* total-energy calculations using a plane-wave basis set. Phys. Rev. B **54**(16), 11169–11186 (1996). <https://doi.org/10.1103/physrevb.54.11169>
3. J.P. Perdew, K. Burke, M. Ernzerhof, Generalized gradient approximation made simple. Phys. Rev. Lett. **77**(18), 3865–3868 (1996). <https://doi.org/10.1103/physrevlett.77.3865>
4. G. Kresse, D. Joubert, From ultrasoft pseudopotentials to the projector augmented-wave method. Phys. Rev. B **59**(3), 1758–1775 (1999). <https://doi.org/10.1103/physrevb.59.1758>
5. P.E. Blöchl, Projector augmented-wave method. Phys. Rev. B **50**(24), 17953–17979 (1994). <https://doi.org/10.1103/physrevb.50.17953>
6. S. Grimme, J. Antony, S. Ehrlich, H. Krieg, A consistent and accurate *ab initio* parametrization of density functional dispersion correction (DFT-D) for the 94 elements H-Pu. J. Chem. Phys. **132**(15), 154104 (2010). <https://doi.org/10.1063/1.3382344>
7. H. Wang, C. Zhu, L. Liu, S. Ma, P. Liu et al., Interfacial residual stress relaxation in perovskite solar cells with improved stability. Adv. Mater. **31**(48), 1904408 (2019). <https://doi.org/10.1002/adma.201904408>
8. J. Tong, J. Gong, M. Hu, S.K. Yadavalli, Z. Dai et al., High-performance methylammonium-free ideal-band-gap perovskite solar cells. Matter **4**(4), 1365–1376 (2021). <https://doi.org/10.1016/j.matt.2021.01.003>
